# Chronic Conductive Hearing Loss Leads to Cochlear Degeneration

**DOI:** 10.1371/journal.pone.0142341

**Published:** 2015-11-18

**Authors:** M. Charles Liberman, Leslie D. Liberman, Stéphane F. Maison

**Affiliations:** 1 Department of Otology and Laryngology, Harvard Medical School, Boston, Massachusetts, United States of America; 2 Eaton-Peabody Laboratory, Massachusetts Eye & Ear Infirmary, Boston, Massachusetts, United States of America; 3 Harvard Program in Speech and Hearing Bioscience and Technology, Boston, Massachusetts, United States of America; University of Washington, Institute for Stem Cells and Regenerative Medicine, UNITED STATES

## Abstract

Synapses between cochlear nerve terminals and hair cells are the most vulnerable elements in the inner ear in both noise-induced and age-related hearing loss, and this neuropathy is exacerbated in the absence of efferent feedback from the olivocochlear bundle. If age-related loss is dominated by a lifetime of exposure to environmental sounds, reduction of acoustic drive to the inner ear might improve cochlear preservation throughout life. To test this, we removed the tympanic membrane unilaterally in one group of young adult mice, removed the olivocochlear bundle in another group and compared their cochlear function and innervation to age-matched controls one year later. Results showed that tympanic membrane removal, and the associated threshold elevation, was counterproductive: cochlear efferent innervation was dramatically reduced, especially the lateral olivocochlear terminals to the inner hair cell area, and there was a corresponding reduction in the number of cochlear nerve synapses. This loss led to a decrease in the amplitude of the suprathreshold cochlear neural responses. Similar results were seen in two cases with conductive hearing loss due to chronic otitis media. Outer hair cell death was increased only in ears lacking medial olivocochlear innervation following olivocochlear bundle cuts. Results suggest the novel ideas that 1) the olivocochlear efferent pathway has a dramatic use-dependent plasticity even in the adult ear and 2) a component of the lingering auditory processing disorder seen in humans after persistent middle-ear infections is cochlear in origin.

## Introduction

Age-related hearing loss is the most common cause of hearing impairment in adults. Loss of threshold sensitivity tends to be bilaterally symmetric, more pronounced at high frequencies, and associated with difficulty in speech discrimination, causing social isolation and cognitive deficits [1–3]. While threshold detection can be improved with hearing aids, gain in intelligibility is often poor, particularly in noisy environments [4]. Even when thresholds are well preserved, speech intelligibility among aged listeners drops in difficult listening environments [5–8].

Loss of cochlear hair cells is a major cause of threshold elevation in age-related hearing loss. In both humans and animals, aging ears show progressive hair cell loss, beginning in the basal, high-frequency end of the cochlear spiral. However, hair cells are not the most vulnerable elements. In both noise-induced [9] and age-related hearing loss [10], the synaptic connections between hair cells and cochlear nerve terminals degenerate first. This primary neural degeneration does not affect thresholds, if diffusely distributed along the cochlea. Indeed, 80% of the cochlear nerve can be lost without threshold elevation, so long as the hair cells are intact [11]. However, this “hidden hearing loss” likely contributes to decreased ability to understand speech, especially in a noisy environment [12].

In aging mice, the number of synaptic connections between cochlear sensory neurons and hair cells decreases by 50% over the 2-year lifespan, and by 25% at middle age, when there is not yet any loss of hair cells [10]. This age-related neuropathy is exacerbated by removing the cochlea’s neuronal feedback, the olivocochlear (OC) bundle: a de-efferented cochlea at middle age looks like a normal cochlea near the end of life [13]. OC feedback comprises two systems: medial (M)OC neurons projecting to outer hair cells, and lateral (L)OC neurons projecting to cochlear nerve terminals in the inner hair cell area [14]. Both systems protect the ear from acoustic overstimulation: MOC fibers by decreasing the outer hair cell’s normal contribution to amplification of sound-induced mechanical vibrations [15], and LOC fibers by reducing glutamate excitotoxicity at the cochlear nerve / hair cell synapses [16].

Hearing thresholds in some non-industrialized societies show minimal deterioration with age, suggesting that cochlear aging is exacerbated by a lifetime of acoustic insults [17–18]. Inspired by these classic human studies, we set out to test whether cochlear neuropathy in normal or de-efferented mice could be minimized, or prevented, by chronically limiting acoustic exposure. Since the ambient noise level in our vivarium is already low, the most effective way to reduce acoustic drive was to surgically remove the eardrum on one side. We observed that one year of conductive hearing loss causes an ipsilateral decrease in LOC innervation and an increase in the age-related loss of afferent synapses. This unanticipated finding suggests significant plasticity of the cochlea’s afferent and efferent innervation, even in the fully developed ear, and is relevant to the treatment of chronic middle-ear infections.

## Materials and Methods

### Animals, Groups and Statistical Analysis

Male CBA/CaJ mice at 6 wks of age were assigned to one of four groups: 1) *Control* animals (n = 11); 2) *OCx* animals (n = 16), in which the olivocochlear (OC) bundle was surgically transected unilaterally and 3) *TMx* animals (n = 10), in which the tympanic membrane (TM) was removed unilaterally. Two additional mice from the *OCx* group were analyzed separately, as they had conductive hearing loss due to otitis media (*OM*) on the side contralateral to the OC transection.

For all groups, mice were anesthetized with xylazine (20 mg/kg, i.p.) and ketamine (100 mg/kg, i.p.). For the *OCx* group, the mouse was held in a stereotaxic apparatus with the scalp retracted. A #11 scalpel blade was lowered into the brain through a skull opening at a position 5.9 mm caudal and 1 mm lateral to the bregma and to a depth of 6.9 mm from the surface. For the *TMx* group, the entire pars tensa of the TM was resected using a right-angle hook. The status of the membrane was re-checked at each cochlear function test day (see below); the resection sometimes had to be repeated, but always became permanent by 16 wks.

After surgery, all mice were returned to the animal care facility. Ambient sound pressure levels inside the cages were previously described [13]. When animal-care personnel were not in the room, sound pressure levels, analyzed in half-octave bands and at 100 msec intervals, were below 40 dB SPL throughout the range of mouse hearing, i.e. from 4 to 64 kHz. Peak noise levels occurred during cage cleaning and daily maintenance, tended to be higher on weekdays than on the weekend and never exceeded 70 dB SPL.

Cochlear function was assessed bilaterally via auditory brainstem responses (ABRs) and distortion product otoacoustic emissions (DPOAEs) at 8, 11, 16, 22, 32, 45 and 64 wks of age. After the final cochlear function test, cochleas were fixed by intracardiac perfusion and removed for histological processing and confocal analysis of hair cell and synaptic degeneration.

All procedures were approved by the IACUC of the Massachusetts Eye and Ear Infirmary. Two-way repeated-measure ANOVAs, adjusted with the Holm-Bonferroni correction, were used to assess the significance of group differences.

### Cochlear Function Tests

For measuring ABRs and DPOAEs, animals were anesthetized with xylazine (20 mg/kg, i.p.) and ketamine (100 mg/kg, i.p.) and placed in an acoustically and electrically shielded room maintained at 32°C. The intertragal notch was slit prior to each electrophysiological recording session to allow stereotyped seating of the acoustic delivery system with a direct and unobstructed path to the eardrum. Acoustic stimuli were delivered through a custom acoustic system consisting of two miniature dynamic earphones used as sound sources (CUI CDMG15008-03A) and an electret condenser microphone (Knowles FG-23329-PO7) coupled to a probe tube to measure sound pressure near the eardrum.

Digital stimulus generation and response processing were handled by digital I-O boards from National Instruments driven by custom LabVIEW software. For ABRs, stimuli were 5-msec tone pips (0.5 msec cos^2^ rise-fall) delivered in alternating polarity at 35/sec. Electrical responses were measured via Grass needle electrodes at the vertex and pinna with a ground reference near the tail and amplified 10,000X with a 0.3–3 kHz passband. Responses to as many as 1024 stimuli were averaged at each sound pressure level, as level was varied in 5 dB steps from below threshold up to 80 dB SPL. For DPOAEs, stimuli were two primary tones f_1_ and f_2_ (f_2_/f_1_ = 1.2), with f_1_ level always 10 dB above f_2_ level. Primaries were swept in 5 dB steps from 20 to 80 dB SPL (for f_2_). The DPOAE at 2f_1_-f_2_ was extracted from the ear canal sound pressure after both waveform and spectral averaging. Noise floor was defined as the average of 6 spectral points below, and 6 above, the 2f_1_-f_2_ point. Threshold was computed by interpolation as the primary level (f_2_) required to produce a DPOAE of 0 dB SPL.

### Cochlear Processing, Immunostaining and Histological Analysis

Mice were perfused intracardially with 4% paraformaldehyde in phosphate buffer. Cochleas were decalcified, dissected into half-turns and permeabilized by freeze/thawing. The half-turns were blocked in 5% normal horse serum (NHS) with 0.3% Triton X-100 (TX) in PBS for 1 hr, followed by incubation for ~19 hrs at 37°C in primary antibodies diluted in 1% NHS with 0.3% TritonX. Primary antibodies included 1) mouse (IgG1) anti-CtBP2 from BD Biosciences at 1:200 to visualize pre-synaptic ribbons, 2) mouse (IgG2a) anti-GluA2, from Millipore at 1:2000 to visualize post-synaptic glutamate receptor patches, and 3) rabbit anti-VAT from Abcam at 1:200 to allow quantification cochlear efferent terminals. Primary incubations were followed by 2 sequential 60-min incubations at 37°C in species-appropriate secondary antibodies with 0.3% TritonX.

Histological analyses were based on high-power confocal z-stacks of the sensory epithelium whole-mounts obtained at half-octave intervals along the cochlear spiral from 5.6 to 64 kHz. To accurately identify regions of interest, cochlear lengths were obtained for each case by tracing the cochlear spiral in low-power images of the dissected pieces using a custom plug-in running under ImageJ (v1.44) that translates cochlear position into frequency using the map for the mouse [19]. Confocal z-stacks were obtained with a glycerol-immersion objective (63X, numerical aperture = 1.3) at 3.17X digital zoom on a Leica TCS SP5. Image spacing in the z plane was set to 0.25 μm, and the z-span was carefully adjusted for each stack to include all synaptic elements in all of the 9–12 hair cells from each row included in each stack, typically requiring ~100 images per stack. Two adjacent stacks were always obtained in each cochlear region sampled.

Four types of information were extracted from inner hair cell and outer hair cell areas in each cochlea: 1) counts of afferent synapses, 2) spatial analysis of afferent synapse locations and inner hair cell alignment along the modiolar-pillar axis, 3) counts of hair cell survival, and 4) the degree of de-efferentation in inner and outer hair cell areas.

1. Pre-synaptic ribbons and post-synaptic glutamate receptor patches in the inner hair cell area were counted from each confocal z-stack using the *connected components* tool in Amira^®^ (Visage Imaging), which finds and displays each voxel space in an image stack containing pixel values greater than a user-set criterion. To quantitatively assess the pairing of pre- and post-synaptic elements, we used custom software that extracts the voxel space within 1 μm around each ribbon (or receptor patch) and produces a thumbnail array of these miniature projections, that can be scanned to count synapses (i.e. ribbons with closely apposed receptor patches) vs. orphan ribbons or orphan receptor patches [20]. The synaptic count in each z-stack was divided by the number of inner hair cells based on counts of their nuclei (including fractional estimates), which stain faintly with anti-CtBP2.
2. To assess the spatial organization of inner hair cell afferent synapses, and the alignment of inner hair cells themselves, we used an approach described in detail elsewhere [21]. Briefly, a user-defined modiolar-pillar axis was superimposed on the zy projection of each z-stack, using custom LabVIEW software. An orthogonal (habenular-cuticular) axis was computed, and the origin defined as the midpoint of the synaptic cloud, along each axis, after transformation to the new coordinate system. Inner hair cell alignment was assessed by measuring the distance between the outermost inner hair cell nuclei along the transformed modiolar-pillar axis.
3. Hair cells in each z-stack were counted by increasing the image output-gain (gamma adjust): Inner hair cell nuclei stain faintly with the CtBP2 antibody, and the outer hair cell somata are visible via their faint background label in several confocal channels, as well as by the presence of synaptic ribbons, even when the efferent terminals are missing.
4. The degree of de-efferentation was assessed in both inner and outer hair cell areas from maximum projections of the VAT-immunostaining in the z-stacks. In both inner and outer hair cell area, the OC innervation was quantified by applying the same threshold algorithm from ImageJ and counting the total number of pixels in maximum projections.

## Results

### Analysis of Cochlear Function and Hair Cell Loss

**Fig 1:**
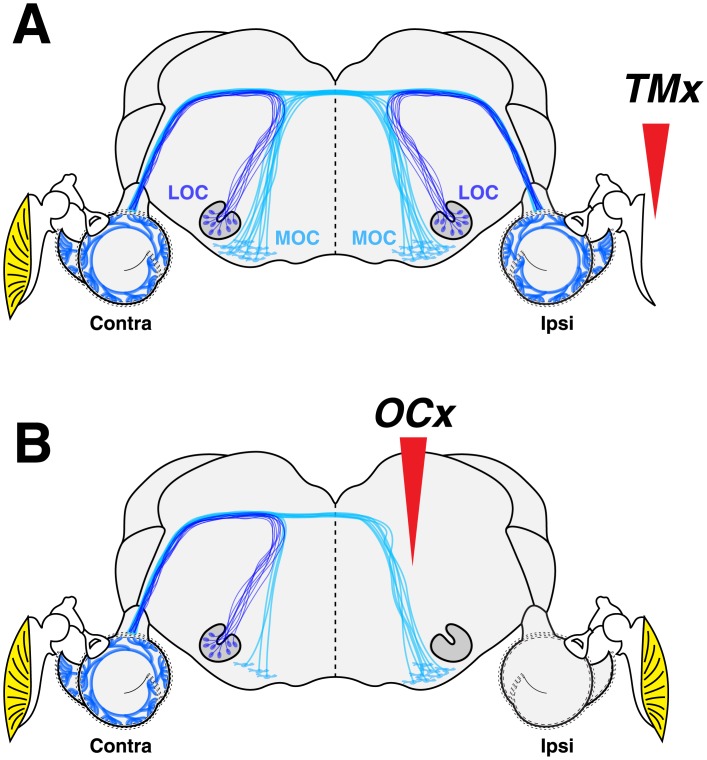
Schematics of the auditory periphery, including the TM, the cochlea and the brainstem locations of the cell bodies of the medial (M) and lateral (L) olivocochlear (OC) neurons. A: Conductive hearing loss was produced by unilateral resection of the TM (*TMx*). B: Cochlear de-efferentation was produced by a stereotaxic section of the olivocochlear bundle in the dorsal surface of the brainstem (*OCx*).

In one group of young (6 wk) mice, we removed most of the TM on one side, and in a second group, we cut the OC bundle on one side (Fig 1). In a third control group, we left everything intact except for a surgical slit at the intertragal notch, which we use in all groups to better visualize the eardrum during cochlear function tests.

**Fig 2:**
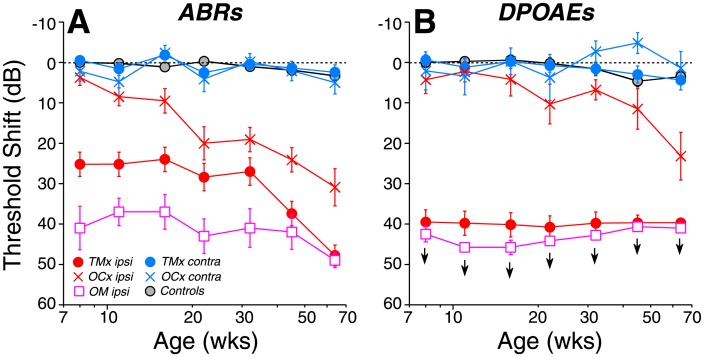
Age-related threshold shifts are exacerbated by ipsilateral OC lesion (*OCx*), TM removal (*TMx*) or otitis media (*OM*). Threshold shift in each group, at each age, was defined *re* mean values at the same test frequency in 8-wk controls. Each point shows mean threshold shift (±SEM) for either ABRs (A) or DPOAEs (B) for frequencies from 5–45 kHz inclusive. Group sizes are: Controls n = 11, *OCx* n = 6; *TMx* n = 10; *OM* n = 2. *OCx* cases were exclusively those where the degree of de-efferentation was greater than 75% at all cochlear regions, as shown in Fig 6. Key in A applies to both panels. Downward arrows indicate that some DPOAE thresholds were at the measurement ceiling, thus the shifts represent a minimum estimate.

Conductive hearing loss was produced by removing the tympanic membrane (TM). The sound attenuation produced by TM perforation depends strongly on the size of the defect [22–23]. Here, TM removal caused an acute threshold elevation of ~25 dB, when measured by ABRs (Fig 2A), a loss comparable to that reported in otitis media [24–25]. Conductive lesions cause larger threshold shifts when measured by DPOAEs (Fig 2B). ABRs, the summed electrical activity of cochlear nerve and auditory brainstem, reflect the reduced sound transmission through the middle ear. However, DPOAEs, which are electrical distortions created, amplified and reverse-transduced back into mechanical vibrations by outer hair cells, are doubly attenuated: first because the reduced sound transmission from ear canal to inner ear reduces the effective stimulus intensity, and second, because reduced transmission of the emissions from the inner ear back to the ear canal reduces the response amplitude [26].

All groups were housed in the vivarium until ~64 wks of age, i.e. ~60% of their average (2.1 yr) lifespan [10], and cochlear function was tested at roughly log-spaced time intervals. Over the first 64 wks of life, there was only a slight (< 3 dB) deterioration of mean ABR and DPOAE thresholds in control ears (Fig 2), as expected from prior study [10]. In the *OCx* ears, age-related threshold deterioration was amplified on the side ipsilateral to the cut, as expected based on prior work showing that cochlear de-efferentation exacerbates both cochlear synaptopathy and hair cell damage [13]. To our surprise, the *TMx* ears, rather than being protected from age-related threshold changes, also showed increased threshold deterioration, especially in the ABR measures, although with a delayed onset relative to the *OCx* ears (Fig 2A). In the *TMx* group, the smaller age-related deterioration of DPOAE thresholds vs. ABR thresholds is consistent with a neural etiology, but this interpretation must be viewed with caution, since DPOAE thresholds immediately after TM removal were already near the ceiling for this measure. The contralateral ears of both *TMx* and *OCx* groups showed minimal age-related changes in cochlear thresholds.

In two of the *OCx* cases, otitis media (OM) developed immediately in the ear opposite the OC lesion, and never resolved. The TM opacity associated with otitis media is clearly visible during cochlear function testing. As seen in Fig 2, these ears also showed significant threshold elevation, as expected from filling of the middle-ear air space with fluid. Interestingly, these two cases also showed increased age-related deterioration of ABR thresholds in the OM ears from 8–64 wks of age, similar to that seen after TM removal (Fig 2A).

**Fig 3:**
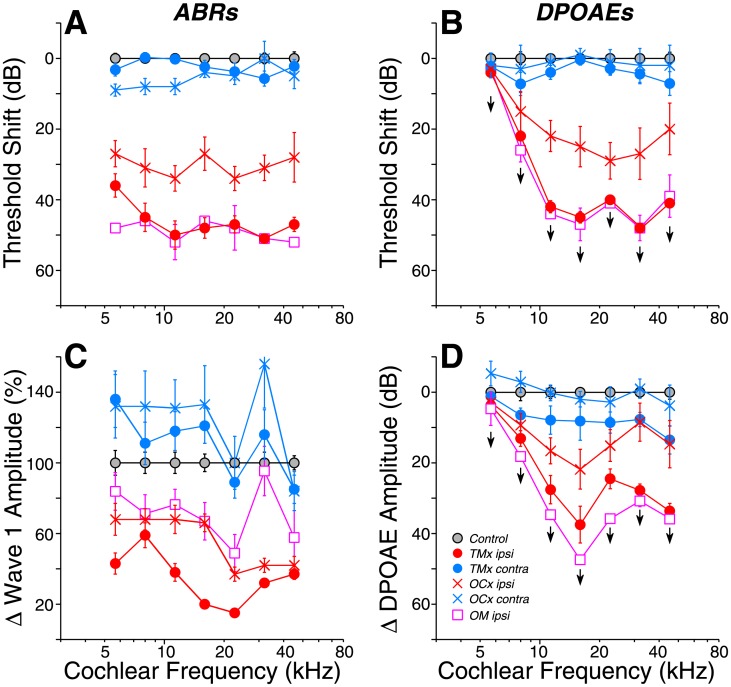
Threshold shifts (A,B) and changes in suprathreshold amplitude (C,D) for ABR (A,C) and DPOAEs (B,D), as measured at 64 wks and normalized to mean age-matched controls. Means (±SEMs) are shown. Amplitudes are computed by extracting the mean response for each ear at each stimulus frequency for stimulus levels from 60–80 dB SPL, inclusive. Groups and group sizes are as defined in Fig 2. Key in C applies to all panels. Downward arrows indicate that the DPOAE thresholds were at or near the measurement ceiling, thus response changes are a minimum estimate.

At 64 wks, the ears with conductive hearing loss showed slightly more ABR threshold elevation at high frequencies (50–60 dB) than at low frequencies (35–45 dB), compared to age-matched controls (Fig 3A). In *OCx* ears, ABR threshold shifts in the ipsilateral ears were flatter with frequency (Fig 3A). At high frequencies, the similarity between DPOAE shifts and ABR shifts in the *OCx ipsi* group suggests that outer hair cell dysfunction underlies the threshold elevation. There is a slight threshold elevation in ears contralateral to either OC lesion or TM removal (Fig 3A and 3B); however, the differences from control were not statistically significant (p > 0.05). The mean suprathreshold amplitudes of ABR wave 1 were larger in the contralateral *TMx* and *OCx* cases (Fig 3C), especially at lower frequencies (5–16 kHz), where the differences *re* controls were statistically significant (p < 0.05 for *OCx* and p < 0.01 for *TMx* ears).

**Fig 4:**
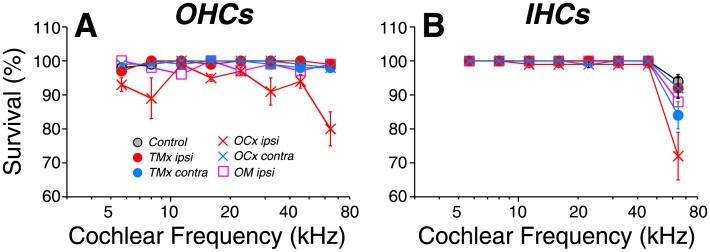
Age-related loss of outer hair cells was increased by OC lesion, but not by chronic conductive hearing loss. Mean survival (± SEMs) of outer hair cells (A) and inner hair cells (B) as a function of cochlear location. Cochleas were harvested at 64 wks. Groups and group sizes are as defined in Fig 2. Key in A also applies to B.

In control ears, there is virtually no loss of either inner or outer hair cells over the first 64 wks of life (Fig 4). Removal of the TM causes no additional hair cell loss. However, cutting the OC bundle causes a small, but statistically significant, increase in outer hair cell loss (p << 0.01), scattered throughout the cochlear spiral (Fig 4A). In none of the groups was there loss of inner hair cells outside of the extreme basal (high-frequency) tip of the cochlea (Fig 4B).

### Analysis of Efferent Innervation Density

**Fig 5:**
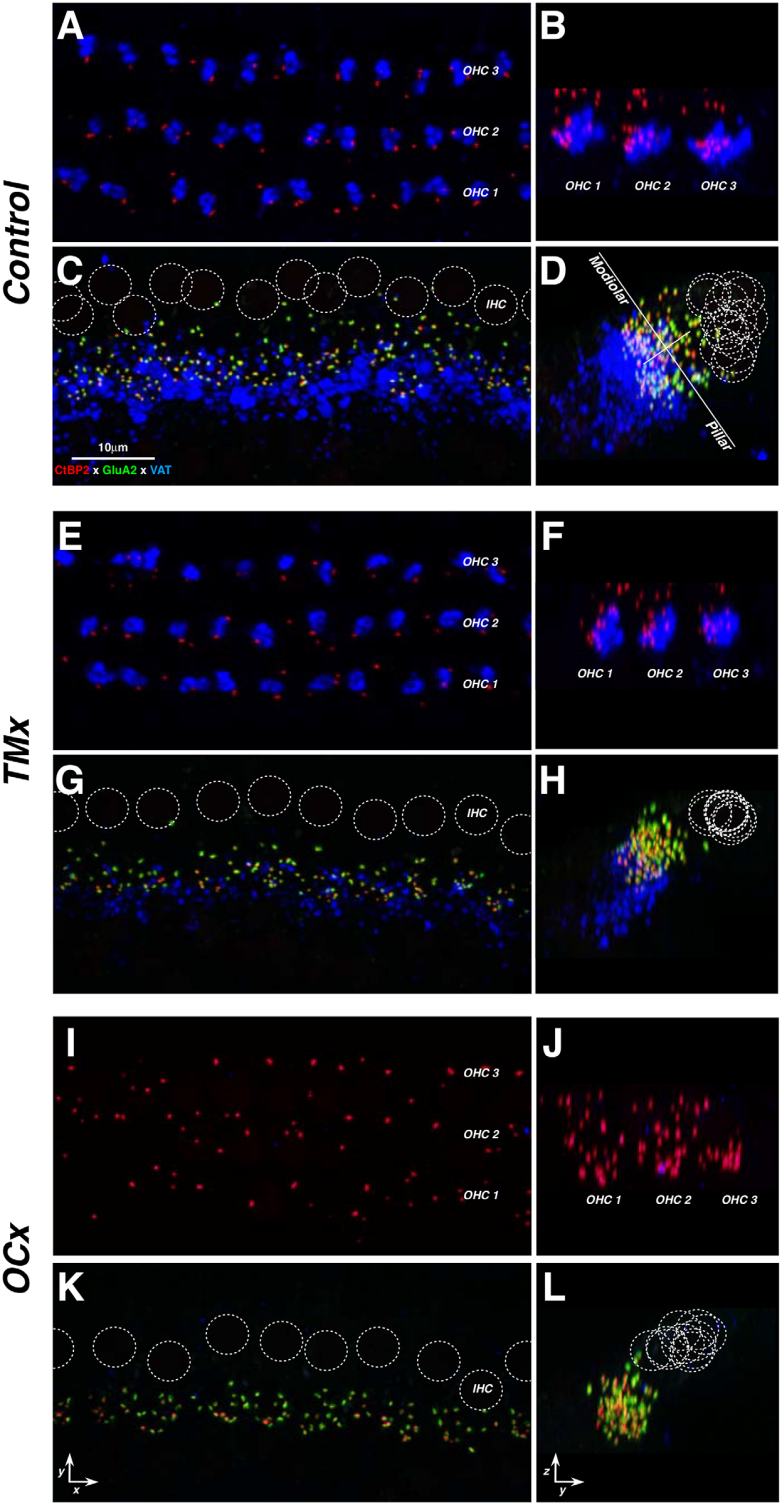
Effects of chronic conductive hearing loss (E,F,G,H) or OC lesion (I,J,K,L) on efferent and afferent innervation in the inner and outer hair cells areas, as compared to control (A,B,C,D). Each row of images (e.g. A,B) contains a pair of maximal projections of a confocal z-stack through 10–12 adjacent hair cells, viewed either in the xy (acquisition) plane (left) or the zy (digitally rotated) plane (right). Each afferent synapse in the inner hair cell area (C,G,K) appears as a closely apposed pair of red (anti-CtBP2) and green (anti-GluA2) puncta. GluA2 puncta are not visible in the outer hair cell area (A,E,I). OC terminals in both inner and outer hair cell areas appear in the blue (anti-VAT) channel. Positions of inner hair cell nuclei are shown as dotted white circles—as seen in the red channel by adjusting gamma (not shown). The white lines in D show the modiolar-pillar and habenular-cuticular axes used in the spatial analysis of inner hair cell synapses (see Fig 9). Orientation of inner hair cells in the zy plane (D,H,L) is as schematized in Fig 9, as are the outer hair cells (B,F,J). Scale in C applies to all micrographs, which are from the 22 kHz region.

To evaluate the OC innervation, we immunostained the cochlear sensory epithelium with a cholinergic marker: vesicular acetylcholine transporter (VAT). As seen in confocal image stacks, each outer hair cell is normally contacted by a cluster of VAT-positive MOC terminals (Fig 5A and 5B). In the inner hair cell area, VAT-positive LOC terminals are smaller and more diffusely organized within the inner spiral bundle, which runs near the basolateral poles of the inner hair cells. The LOC terminals make synaptic contacts primarily with the dendrites of cochlear nerve terminals near their hair cell synapses [27].

**Fig 6:**
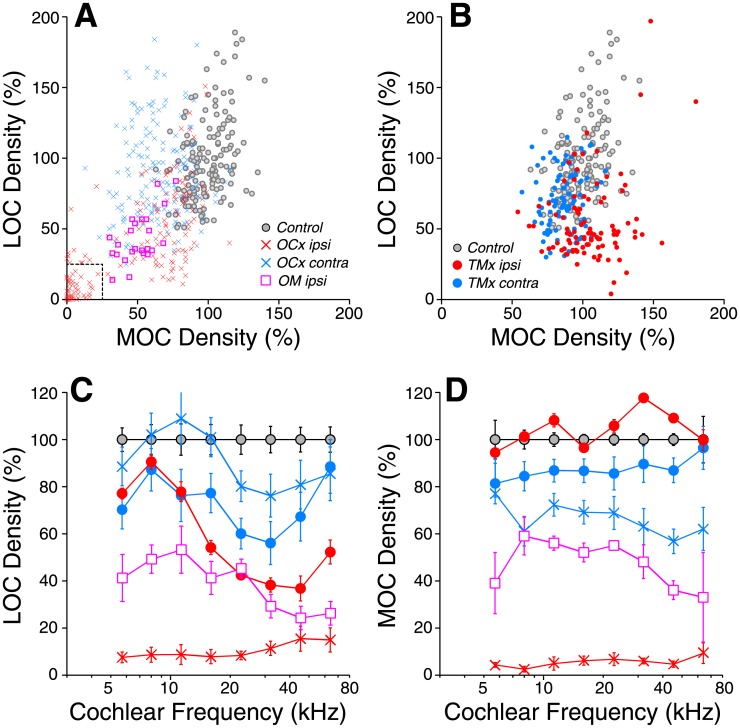
Chronic conductive hearing loss, by either TM removal or otitis media, reduces OC innervation, though not as completely as can be achieved by OC lesion. A,B: Comparison of LOC and MOC density in cochlear regions ipsilateral (red, magenta) or contralateral (blue) to olivocochlear lesion (*OCx*) or otitis media (*OM*) (A) or tympanic membrane removal (*TMx*) (B). Each point is a different cochlear region from a different case (8–45 kHz regions are shown). Data from each cochlear region are normalized to mean values from age-matched controls (gray). Dashed box in A delineates 75% de-efferentation for both MOC and LOC: only cases for which all cochlear regions are within that box are included in the *OCx* group. C,D: Mean LOC and MOC innervation densities (± SEMs), respectively, as a function of cochlear frequency for the different experimental groups, normalized to control means. Keys in A and B also apply to C and D.

When accurately positioned, brainstem cuts (Fig 1B) can completely eliminate LOC and MOC projections to the ipsilateral ear, as illustrated in Fig 5I and 5L, and as shown more quantitatively in Fig 6A, 6C and 6D. In 8 out of 16 cases, survival of LOC and MOC terminals was < 25% at all cochlear locations (Fig 6A). We defined this subset as the *OCx* cases. As shown in Fig 6C and 6D, the de-efferentation in this subgroup was similarly effective throughout the cochlear spiral. The small but significant decrease in OC innervation in the contralateral ears (p << 0.01 for MOC; p << 0.01 for LOC at frequencies above 22 kHz) could arise because a small fraction of OC neurons project bilaterally [14] and undergo retrograde degeneration after severing the axonal projection to one ear. The larger contralateral effect on the MOC system may arise because the cut is deep enough to sever some of the contralaterally projecting MOC axons in the ipsilateral olivary complex (see Fig 1B).

Unexpectedly, the *TMx* cases also showed significant ipsilateral decrease in LOC innervation density (Fig 6C, p << 0.01). That de-efferentation was especially pronounced in the basal half of the cochlea, where the LOC density was less than half of that seen in control ears (Fig 6C). In contrast, the mean MOC innervation density was not significantly different from control (p > 0.05). The possible relevance of this observation to cases of chronic middle ear infection is underscored by data from the two cases with otitis media. As shown in Fig 6C, the loss of LOC innervation in the *OM ipsi* cases is similar to that seen in the *TMx* ears. The loss of MOC innervation is very different from the *TMx ipsi* ears, but similar to the *OCx contra* ears, likely because the *OM* cases also had an OC cut on the opposite side.

### Analysis of Afferent Innervation Density

At each synapse between a hair cell and a cochlear nerve fiber, a pre-synaptic ribbon in the hair cell apposes a post-synaptic density on the nerve terminal [28–29]. Thus, when the organ of Corti is immunostained with antibodies against a ribbon protein (CtBP2 [30]), and an AMPA-type glutamate receptor subunit (GluA2), each afferent synapse is seen as a pair of puncta (Fig 5C). Outer hair cell synapses (Fig 5A) do not immunostain with anti-GluA2 in the adult ear [20]. Here, we concentrate on the inner hair cell area, because all of the myelinated (fast-conducting) cochlear nerve fibers synapse there [31], and because the functional significance of the small population of unmyelinated sensory fibers contacting the outer hair cells is very poorly understood [32].

**Fig 7:**
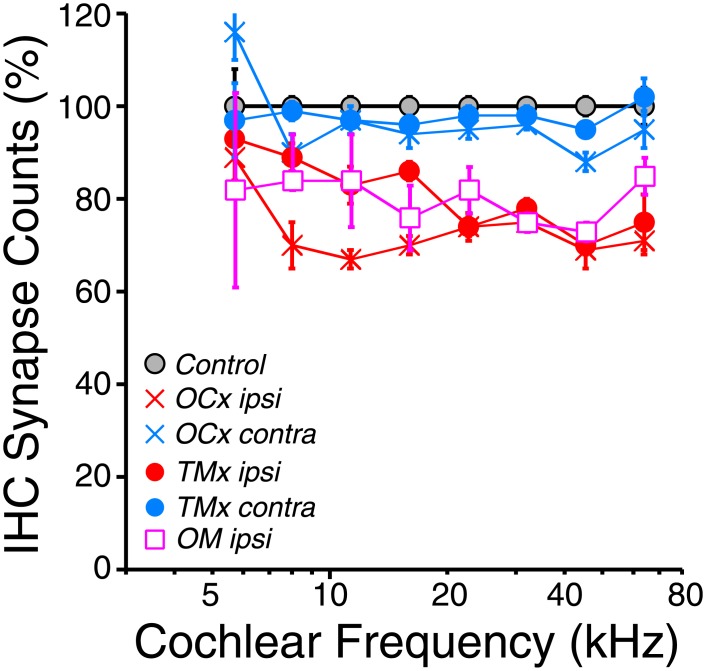
Inner hair cell synaptic counts are reduced in ears ipsilateral to either OC lesion, TM removal or chronic otitis media. For each group, mean synaptic counts (± SEMs) were normalized to means of age-matched controls (64 wks). Group sizes are as defined in Fig 2.

In the young adult mouse, there are from 10–18 cochlear-nerve synapses per inner hair cell, depending on cochlear location [20,33]. In the normally aging mouse, roughly 25% of those synapses disappear by 64 wks [10]. Prior work showed that this age-related cochlear neuropathy increases significantly in partially de-efferented cochleas [13]. Here, we observed that de-efferentation accelerates cochlear neuropathy, whether the de-efferentation was due to cutting the OC bundle (p << 0.01) or a chronic conductive hearing loss, and whether the conductive hearing loss was by TM removal (p << 0.01) or otitis media (p << 0.01) (Fig 7). The data in Fig 6 suggest that the condition of the LOC system is most relevant to the development of afferent synaptopathy. *TMx ipsi* cases show increasing LOC loss from apex to base (Fig 6C), and a similar gradient of afferent synaptopathy (Fig 7). The condition of the MOC innervation appears unrelated to the afferent synaptopathy: MOC innervation was normal in *TMx ipsi* cases and *OCx contra* cases show no significant synaptic loss (p > 0.05), despite MOC loss approaching 40% in some cochlear regions (Fig 6D).

**Fig 8:**
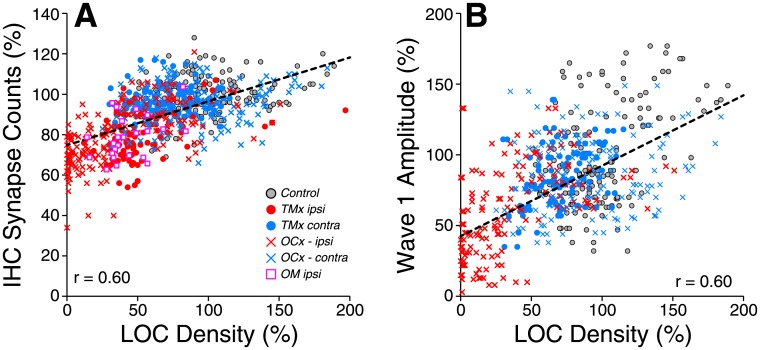
The relation between the degree of de-efferentation in the inner hair cell area and loss of afferent synapses (A) or ABR Wave 1 amplitude (B) is similar whether the de-efferentation is caused by OC lesion or by chronic conductive hearing loss. Data include all frequency regions from 8 to 45 kHz. Data are normalized to the mean value for age-matched controls at the appropriate frequency region. Wave 1 amplitudes (B) are extracted for stimulus levels from 60–80 dB SPL, as in Fig 3. Data are not shown for *TMx ipsi* and *OM ipsi* groups since conductive hearing loss, *per se* profoundly decreases ABR amplitudes. Groups and group sizes are as defined in Fig 2. Key in A also applies to B.

When examined on a case-by-case basis, there was a correlation between the degree of LOC loss in a particular cochlear region and the degree of synaptic loss in the same confocal z-stack (Fig 8A: r = 0.60). There was a similar correlation between degree of LOC de-efferentation and the decrement in maximum amplitude of ABR wave 1 in the same case and frequency region (Fig 8B: r = 0.60).

### Analysis of Spatial Patterning in the Inner Hair Cell Area

Cochlear nerve fibers are divided into functional subgroups with spatial polarization of their hair cell contacts: high-threshold fibers with low spontaneous rates (SRs) synapse on the side of the inner hair cell closer to the modiolus, while low-threshold fibers with high SRs synapse on the side of the hair cell closer to the pillar cells [28]. Since low-SR fibers are more vulnerable to age and noise [34–35], we analyzed the spatial patterning of synapses along the modiolar-pillar axis.

**Fig 9:**
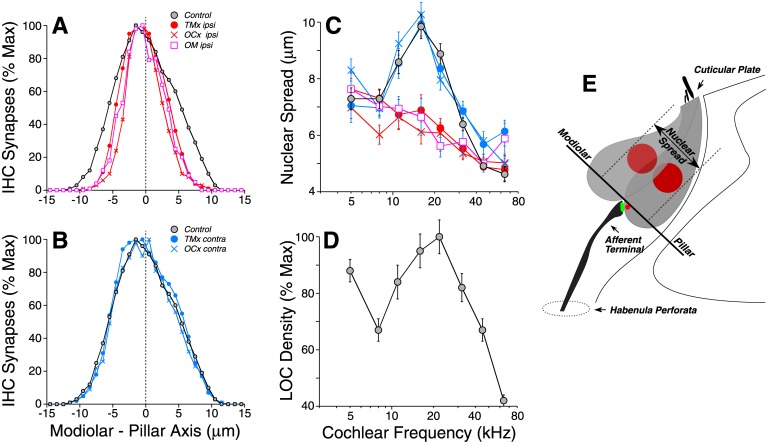
Loss of efferent innervation, whether by OC lesion or chronic conductive hearing loss, compresses the spatial distribution of afferent synapses (A,B), and inner hair cells (C), along the modiolar-pillar axis; the staggered packing of adjacent hair cells, which arises from hair cell hypertrophy, is normally maximal (C) at cochlear locations where the LOC innervation is densest (D). A,B: Spatial distribution of inner hair cell afferent synapses along the modiolar-pillar axis for each experimental groups compared to age-matched controls. Synaptic location is defined as described in Methods. Mean values are shown for each group within each 1-μm location bin. Data are combined across all cochlear locations. C: Staggering of the inner hair cell row was quantified by measuring the nuclear spread (see schematic in E), as seen in yz projections of confocal z-stacks (Fig 5). Mean values (±SEMs) are shown. D: Mean density of LOC innervation (±SEMs) in age-matched control ears as a function of cochlear location. Symbol keys in A,B also apply to C,D. E: Schematic illustrating the staggered positioning of inner hair cell nuclei along the modiolar pillar axis, and the measure of “nuclear spread” used to quantify it.

As seen in Fig 5D, the synapses under the inner hair cells are normally found in a cloud that is elongated along the modiolar-pillar axis. Prior studies show that modiolar synapses have larger ribbons and smaller GluA patches than pillar synapses [20]. Here, qualitative analysis suggested that this elongate cloud collapsed along the modiolar-pillar axis in de-efferented ears, whether the de-efferentation was caused by OC lesion or by conductive hearing loss (See Fig 5H and 5L). A more quantitative analysis of the spread of synapses along the modiolar-pillar axis clearly showed the trend towards a more compact cloud in the de-efferented ears (Fig 9A), with no change from age-matched controls in the ears contralateral to the de-efferentation (Fig 9B).

In prior work [21], we noted that the elongation of the normal synaptic cloud arose, at least in part, because adjacent hair cells take up staggered positions, when viewed along the hair cell row, presumably to increase packing density of these flask-shaped cells, which are larger in diameter at their basolateral poles than at their cuticular plates (Fig 9E). In the de-efferented ears, qualitative analysis suggested that this alternating alignment of adjacent hair cells largely disappeared: compare the compact distribution of inner hair cell nuclei in Fig 5H and 5L to the diffuse distribution in the normal ear, Fig 5D. Again, a more quantitative analysis clearly showed this difference (Fig 9C): de-efferented ears show a compact nuclear alignment, while the contralateral ears look like age-matched controls. It may not be a coincidence that, in the normal ear, there is a correlation between the apex-base pattern of LOC innervation density (Fig 9D) and the apex-base pattern of nuclear spread (Fig 9C). One interpretation of these trends is that a dense LOC innervation enhances overall metabolic rate in inner hair cells, which increases their diameters, which, in turn, increases nuclear misalignment. Correspondingly, a loss of efferent innervation causes inner hair cell hypotrophy and thereby increases the alignment of adjacent nuclei.

## Discussion

### OC-mediated protection against age-related neuropathy and hair cell death

Rather than providing protection by reducing the acoustic drive to the inner ear, a chronic conductive hearing loss induced by TM removal increased cochlear-nerve synaptopathy in the ipsilateral ear. The loss of hair cell synapses was as dramatic as that seen after complete surgical de-efferentation (Fig 7). Furthermore, a conductive hearing loss of 1-yr duration also reduced the density of OC terminals in the cochlear epithelium, suggesting that the loss of efferent feedback, *per se*, was the common cause of the cochlear synaptopathy (Fig 6). The further observation that otitis media caused similar cochlear de-efferentation and cochlear-nerve synaptopathy to that seen after TM removal (Fig 6) strongly suggests that it is the loss of acoustic drive that caused the changes in cochlear efferent and afferent innervation densities, rather than some other effect of the TM surgery, such as transient acoustic trauma from manipulation of the eardrum. Further evidence is provided by the demonstration that complete TM removal in mouse causes minimal changes in bone-conduction thresholds, even when coupled with the more traumatic procedure of removing the malleus [36]. The fact that all experimental groups underwent anesthetization and minor pinna surgery on each cochlear-function test day (see Methods) strongly suggests that it is the TM removal that caused the cochlear degeneration, rather than some less specific effect of anesthetization or surgical stress: the TM removal maneuver itself is exceedingly brief.

The present results also suggest that it is the LOC, rather than the MOC, division of the cochlea’s efferent feedback network that modulates the survival of cochlear nerve synapses in the aging ear. Since the (unmyelinated) LOC and the (myelinated) MOC fibers run intermingled within the OC bundle, surgical transection typically removes both types of efferent fibers in roughly equal proportion. Chronic conductive hearing loss, in contrast, affected only the density of LOC terminals (Fig 6). Since TM removal causes synaptopathy comparable to that seen with OC bundle transection, the strong implication is that the synaptic protection is LOC mediated. The LOC system contains two subtypes, one cholinergic and one dopaminergic [37]. The dopaminergic population has been implicated in the neuro-protective effect: application of dopamine agonists can reduce spontaneous and sound-evoked discharge rates in cochlear nerve fibers, and perfusion of dopamine agonists through the cochlea during acoustic overexposure can reduce the prominent swelling of cochlear nerve terminals seen at short post-exposure survival times [16]. However, in mouse cochlea, the dopaminergic innervation is sparse [37], and thus it is difficult to use dopaminergic markers to assess the degree of de-efferentation.

Data obtained in this, and a prior, study suggest that LOC-mediated synaptic protection requires a “threshold level” of roughly 50% of the normal innervation density, above which, the synapses are protected, and below which some population of cochlear nerve synaptic terminals is at risk. In the present study, TM removal and OC bundle transection caused similar degrees of cochlear synaptopathy in the basal half of the cochlea (Fig 7), yet the TM removal caused only 50–60% loss of LOC terminals, whereas the OC bundle transection removed > 90% of the LOC innervation (Fig 6). A prior study of OC bundle transection, using a different surgical approach, achieved a less complete degree of de-efferentation. However, being just over the putative “threshold” of 50% in most cochlear regions, this partial de-efferentation also enhanced cochlear synaptopathy [13]. Conversely, the ears contralateral to TM removal in the present study showed only 40% loss of LOC terminals, and correspondingly did not demonstrate any significant cochlear synaptopathy compared to age-matched controls (Figs 6 and 7).

The loss of LOC innervation was also correlated with hypotrophy of the inner hair cells, manifested as an increased alignment of adjacent nuclei when viewed along the cochlea’s spiral axis (Fig 9A). Although this hypotrophy could arise directly from the chronic decrease in acoustic stimulation, the coincidence of the normal LOC density peak with the normal peak in inferred hair cell hypertrophy (Fig 9C and 9D) suggests that the changes in hair cell size are driven by the LOC innervation, perhaps via the small population of direct synapses between LOC terminals and inner hair cells [27], or perhaps indirectly via their synapses on cochlear nerve terminals.

MOC terminals are virtually all cholinergic [38–39], and their activation inhibits the outer hair cells and reduces their normal contribution to cochlear amplification. MOC fibers are responsive to sound and thus form the effector arm of a sound-evoked negative feedback loop that reduces sound-evoked mechanical motions of the cochlear partition [40]. Present results are consistent with many prior reports suggesting that this negative feedback loop can protect the outer hair cells from acoustic overstimulation [41–42], except in the present study the only acoustic stimulation present was the everyday noise of the animal vivarium. Here we show that outer hair cell survival is modestly jeopardized in ears with loss of the MOC terminals subsequent to OC bundle transection (Fig 4). Since outer hair cell survival is key to the maintenance of normal thresholds, these results are consistent with implications from human studies suggesting that age-related thresholds shifts are largely due to the accumulation of a lifetime of exposure to everyday sounds in our overly noisy modern environments [17]. They further support the idea that some of the inter-subject variability in vulnerability to age-related hearing loss may arise from individual differences in the strength of the sound-evoked MOC reflex [43].

### Conductive hearing loss and the development of cochlear pathology

Sensory deprivation has long-lasting, deleterious effects on brain and behavior. In studies of the auditory system, disruption of the middle-ear has been a common manipulation used to acoustically deprive one ear. Most such studies have imposed the conductive hearing loss during the neonatal period [44–46] and most have evaluated the effects at the level of the auditory brainstem and cortex [47–48], rather than in the cochlea. There are many dramatic effects of acoustic deprivation on neuronal morphology and responses, especially when the deprivation occurs during a critical developmental period [49–51].

Here, we introduced acoustic deprivation at 6 wks of age, a time in the mouse when the ear appears mature in form, as seen in histological analysis at the light microscopic level, and in function, at least as far as can be observed by measuring thresholds by ABRs and DPOAEs [10]. Studies of unilateral conductive hearing loss of only a few weeks duration in adult guinea pigs reported dramatic changes to the mix of excitation and inhibition in cochlear nucleus responses, consistent with the view that the contralateral pathways increase activation to compensate for the ipsilateral threshold elevation [52]. Such a view is consistent with hints in the present study of elevated ABR Wave 1 amplitudes in the ears contralateral to TM removal (Fig 3C).

In prior work, we suggested that one function of the LOC system was to binaurally balance excitability of the left and right cochlear nerves in the face of changing sound transmission, due, for example to a unilateral conductive hearing loss [53]. Such binaural balance must be important in maintaining accuracy in sound localization that requires calculations based on interaural intensity differences. A prediction of that model is that unilateral conductive hearing loss should modulate cochlear nerve excitability in the opposite ear, hints of which were observed here (Fig 3C). A prior study failed to observe such an effect, but that study only waited 1 month after TM removal [54]. Of course, in the proposed model, the modulation of auditory nerve activity was supposed to occur by changing the balance of LOC-mediated excitation and inhibition in the cholinergic and dopaminergic pathways, respectively, not by loss of LOC peripheral terminals, as observed here. Furthermore, in the present study, the changes in LOC innervation were not effective in counteracting the disruptive effects of the conductive hearing loss; indeed, the resultant partial de-efferentation appears to have exacerbated the loss of cochlear nerve synapses.

At least 80% of children will experience one or more bouts of otitis media before they reach 3 years of age, making it the most common cause for physician visits and medication prescriptions among children in the USA [55]. These bouts can persist for many months in some cases, and deficits in spatial hearing as well as receptive language skills can persist for years after the middle-ear pathology has resolved [56]. Several human studies suggest that chronic conductive hearing loss can slowly develop a sensorineural component as well [57–61]; however the only relevant human histopathological study concentrates on cases where a persistent bacterial infection in the middle ear appears to have spread to the inner ear, causing hair cell damage [62]. Data from the present study suggest that the auditory deprivation, *per se*, damages the efferent and afferent innervation of the hair cells, in ways similar to that seen in age-related and noise-induced hearing loss. Although the mechanisms underlying cochlear de-efferentation following sound-deprivation are not known, its effects need to be considered in the management of chronic conductive hearing loss in clinic.

## References

[1] Mick P, Kawachi I, Lin FR. The association between hearing loss and social isolation in older adults. Otolaryngol Head Neck Surg. 2014;150: 378–84. 10.1177/0194599813518021 24384545

[2] Deal JA, Sharrett AR, Albert MS, Coresh J, Mosley TH, Knopman D, Wrick LM, Lin FR. Hearing impairment and cognitive decline: a pilot study conducted within the atherosclerosis risk in communities neurocognitive study. Am J Epidemiol. 2015; 181: 680–90. 10.1093/aje/kwu333 25841870 PMC4408947

[3] Genther DJ, Betz J, Pratt S, Kritchevsky SB, Martin KR, Harris RB, et al Association of hearing impairment and mortality in older adults. J Gerontol A Biol Sci Med Sci. 2015;70: 85–90. 10.1093/gerona/glu094 25024235 PMC4296166

[4] Van Eyken E, Van Camp G, Van Laer L. The complexity of age-related hearing impairment: contributing environmental and genetic factors. Audiol Neurootol. 2007;12: 345–58. 17664866 10.1159/000106478

[5] Dubno JR, Dirks DD, Morgan DE. Effects of age and mild hearing loss on speech recognition in noise. J Acoust Soc Am. 1984;76: 87–96. 6747116 10.1121/1.391011

[6] Snell KB, Frisina DR. Relationships among age-related differences in gap detection and word recognition. J Acoust Soc Am. 2000;107: 1615–26. 10738815 10.1121/1.428446

[7] Walton JP. Timing is everything: temporal processing deficits in the aged auditory brainstem. Hear Res. 2010;264: 63–9. 10.1016/j.heares.2010.03.002 20303402 PMC7045868

[8] Ruggles D, Bharadwai H, Shinn-Cunningham BG. Why middle-aged listeners have trouble hearing in everyday settings. Curr Biol. 2012;22: 1417–22. 10.1016/j.cub.2012.05.025 22727697 PMC3756149

[9] Kujawa SG, Liberman MC. Adding insult to injury: cochlear nerve degeneration after "temporary" noise-induced hearing loss. J Neurosci. 2009;29: 14077–85. 10.1523/JNEUROSCI.2845-09.2009 19906956 PMC2812055

[10] Sergeyenko Y, Lall K, Liberman MC, Kujawa SG. Age-related synatopathy: an early-onset contributor to auditory functional decline. J Neurosci. 2013;33: 13686–94. 10.1523/JNEUROSCI.1783-13.2013 23966690 PMC3755715

[11] Schuknecht HF, Woellner RC. An experimental and clinical study of deafness from lesions of the cochlear nerve. J Laryngol Otol. 1955;69: 75–97. 14354340 10.1017/s0022215100050465

[12] Kujawa SG, Liberman MC. Synaptopathy in the noise-exposed and aging cochlea: Primary neural degeneration in acquired sensorineural hearing loss. Hear Res. 2015; in press. 10.1016/j.heares.2015.02.009 PMC4567542 25769437

[13] Liberman MC, Liberman LD, Maison SF. Efferent feedback slows cochlear aging. J Neurosci. 2014;34: 4599–607. 10.1523/JNEUROSCI.4923-13.2014 24672005 PMC3965785

[14] Warr WB, Guinan JJ, White JS. Organization of the efferent fibers: the lateral and medial olivocochlear systems Neurobiology of hearing: the cochlea. Altschuler RA, Hoffman DW, editors. New York: Raven; 1986.

[15] Guinan JJ Jr. Cochlear efferent innervation and function. Curr Opin Otolaryngol Head Neck Surg. 2010;18: 447–53. 10.1097/MOO.0b013e32833e05d6 20717032 PMC3075443

[16] Ruel J, Wang J, Rebillard G, Eybalin M, Lloyd R, Pujol R, et al Physiology, pharmacology and plasticity at the inner hair cell synaptic complex. Hear Res. 2007;227: 19–27. 17079104 10.1016/j.heares.2006.08.017

[17] Rosen S, Bergman M, Plester D, El-Mofty A, Satti MH. Presbycusis study of a relatively noise-free population in the Sudan. Ann Otol Rhinol Laryngol. 1962;71: 727–43. 13974856 10.1177/000348946207100313

[18] Goycoolea MV, Goycoolea HG, Farfan CR, Rodriguez LG, Martinez GC, Vidal R. Effect of life in industrialized societies on hearing in natives of Easter Island. Laryngoscope. 1986; 96: 1391–6. 3784745 10.1288/00005537-198612000-00015

[19] Taberner AM, Liberman MC. Response properties of single auditory nerve fibers in the mouse. J Neurophysiol. 2005;93: 557–69. 15456804 10.1152/jn.00574.2004

[20] Liberman LD, Wang H, Liberman MC. Opposing gradients of ribbon size and AMPA receptor expression underlie sensitivity differences among cochlear-nerve/hair-cell synapses. J Neurosci. 2011;31: 801–8. 10.1523/JNEUROSCI.3389-10.2011 21248103 PMC3290333

[21] Yin Y, Liberman LD, Maison SF, Liberman MC. Olivocochlear innervation maintains the normal modiolar-pillar and habenular-cuticular gradients in cochlear synaptic morphology. J Assoc Res Otolaryngol. 2014;15: 571–83. 10.1007/s10162-014-0462-z 24825663 PMC4141434

[22] Voss SE, Rosowski JJ, Merchant SN, Peake WT. Middle-ear function with tympanic-membrane perforations. I. Measurements and mechanisms. J Acoust Soc Am. 2001;110:1432–44. 11572354 10.1121/1.1394195

[23] Mehta RP, Rosowski JJ, Voss SE, O’Neil E, Merchant SN. Determinants of hearing loss in perforations of the tympanic membrane. Otol Neurotol. 2006;27:136–43. 16436981 10.1097/01.mao.0000176177.17636.53 PMC2918411

[24] Kokko E. Chronic secretory otitis media in children. A clinical study. Acta Otolaryngol Suppl. 1974;327:1–44. 4534033

[25] Gravel JS, Roberts JE, Roush J, Grose J, Besing J, Burchinal M, Neebe E, Wallace IF, Zeisel S. Early otitis media with effusion, hearing loss, and auditory processes at school age. Ear Hear. 2006;27:353–68. 16825885 10.1097/01.aud.0000224727.45342.e9

[26] Qin Z, Wood M and Rosowski JJ. Measurement of conductive hearing loss in mice. Hearing Res. 2010; 253(1–2):93–103 10.1016/j.heares.2009.10.002 PMC2866764 19835942

[27] Liberman MC. Efferent synapses in the inner hair cell area of the cat cochlea: an electron microscopic study of serial sections. Hear Res. 1980;3: 189–204. 7440423 10.1016/0378-5955(80)90046-5

[28] Liberman MC. Morphological differences among radial afferent fibers in the cat cochlea: An electron-microscopic study of serial sections. Hear Res. 1980;3: 45–63. 7400048 10.1016/0378-5955(80)90007-6

[29] Khimich D, Nouvian R, Pujol R, Tom Dieck S, Egner A, Gundelfinger ED, et al Hair cell synaptic ribbons are essential for synchronous auditory signaling. Nature. 2005;434: 889–94. 15829963 10.1038/nature03418

[30] Schmitz F, Königstorfer A, Südhof TC. A component of synaptic ribbons: a protein’s journey through evolution provides insight into synaptic ribbon function. Neuron. 2000;28: 857–72. 11163272 10.1016/s0896-6273(00)00159-8

[31] Liberman MC. Single-neuron labeling in the cat auditory nerve. Science. 1982;216: 1239–41. 7079757 10.1126/science.7079757

[32] Flores EN, Duggan A, Madathany T, Hogan AK, Marques F, Kumar G, et al A non-canonical pathway from cochlea to brain signals tissue-damaging noise. Current Biology. 2015; 25: 606–12. 10.1016/j.cub.2015.01.009 25639244 PMC4348215

[33] Meyer AC, Frank T, Khimich D, Hoch G, Riedel D, Chapochnikow NM, et al Tuning of synapse number, structure and function in the cochlea. Nat Neurosci. 2009;12: 444–53. 10.1038/nn.2293 19270686

[34] Schmiedt RA, Mills JH, Boettcher FA. Age-related loss of activity of auditory-nerve fibers. J Neurophysiol. 1996;76: 2799–803. 8899648 10.1152/jn.1996.76.4.2799

[35] Furman AC, Kujawa SG, Liberman MC. Noise-induced cochlear neuropathy is selective for fibers with low spontaneous rates. J Neurophysiol. 2013;110: 577–86. 10.1152/jn.00164.2013 23596328 PMC3742994

[36] Chhan D, McKinnon M. Rosowski JJ. Identification of conductive hearing loss in CBA/CaJ mice using bone conduction. Abstract #454 of the 36^th^ Annual Midwinter Meeting of the Association for Research in Otologyngology. 2013;36: 66.

[37] Darrow KN, Simons EJ, Dodds L, Liberman MC. Dopaminergic innervation of the mouse inner ear: evidence for a separate cytochemical group of cochlear efferent fibers. J Comp Neurol. 2006;498: 403–14. 16871528 10.1002/cne.21050 PMC1805779

[38] Rajan R. Effect of electrical stimulation of the crossed olivocochlear bundle on temporary threshold shifts in auditory sensitivity. II. Dependence on the level of temporary threshold shifts. J Neurophysiol. 1988;60: 569–79. 3171642 10.1152/jn.1988.60.2.569

[39] Maison SF, Adams JC, Liberman MC. Olivocochlear innervation in the mouse: immunocytochemical maps, crossed versus uncrossed contributions, and transmitter colocalization. J Comp Neurol. 2003;455: 406–16. 12483691 10.1002/cne.10490 PMC1805785

[40] Guinan JJ Jr. Physiology of olivocochlear efferents The cochlea. Dallos P, Popper AN, Fay RR, editors. New York: Springer; 1996 p. 435–502.

[41] Cody AR, Johnstone BM. Reduced temporary and permanent hearing losses with multiple tone exposures. Hear Res. 1982;6: 291–301. 7085486 10.1016/0378-5955(82)90061-2

[42] Maison SF, Luebke AE, Liberman MC, Zuo J. Efferent protection from acoustic injury is mediated via alpha9 nicotinic acetylcholine receptors on outer hair cells. J Neurosci. 2002;22: 10838–46. 12486177 10.1523/JNEUROSCI.22-24-10838.2002 PMC6758430

[43] Maison SF, Liberman MC. Predicting vulnerability to acoustic injury with a noninvasive assay of olivocochlear reflex strength. J Neurosci. 2000;20: 4701–7. 10844039 10.1523/JNEUROSCI.20-12-04701.2000 PMC6772446

[44] Smith ZD, Gray L, Rubel EW. Afferent influences on brainstem auditory nuclei of the chicken: n. laminaris dendritic length following monaural conductive hearing loss. J Comp Neurol. 1983;220: 199–205. 6315783 10.1002/cne.902200207

[45] Tucci DL, Rubel EW. Afferent influences on brain stem auditory nuclei of the chicken: effects of conductive and sensorineural hearing loss on n. magnocellularis. J Comp Neurol. 1985;238: 371–81. 4044922 10.1002/cne.902380402

[46] Tucci DL, Born DE, Rubel EW. Changes in spontaneous activity and CNS morphology associated with conductive and sensorineural hearing loss in chickens. Ann Otol Rhinol Laryngol. 1987;96: 343–50. 3605960 10.1177/000348948709600321

[47] Kandler K, Gillepsie DC. Developmental refinement of inhibitory sound-localization circuits. Trends Neurosci. 2005;28: 290–6. 15927684 10.1016/j.tins.2005.04.007 PMC4120098

[48] Dahmen JC, King AJ. Learning to hear: plasticity of auditory cortical processing. Curr Opin Neurobiol. 2007;17: 456–64. 17714932 10.1016/j.conb.2007.07.004

[49] Coleman JR, O’Connor P. Effects of monaural and binaural sound deprivation on cell development in the anteroventral cochlear nucleus of rats. Exp Neurol. 1979;64: 553–66. 467549 10.1016/0014-4886(79)90231-0

[50] Webster DB, Webster M. Effects of neonatal conductive hearing loss on brain stem auditory nuclei. Ann Otol Rhinol Laryngol. 1979;88: 684–8. 496200 10.1177/000348947908800515

[51] Conlee JW, Parks TN. Late appearance and deprivation-sensitive growth of permanent dendrites in the avian cochlear nucleus (nuc. magnocellularis). J Comp Neurol. 1983;217: 216–26. 6886053 10.1002/cne.902170208

[52] Sumner CJ, Tucci DL, Shore DE. Responses of ventral cochlear nucleus neurons to contralateral sound after conductive hearing loss. J Neurophysiol. 2005;94: 4234–43. 16093339 10.1152/jn.00401.2005

[53] Darrow KN, Maison SF, Liberman MC. Cochlear efferent feedback balances interaural sensitivity. Nat Neurosci. 2006;9: 1474–6. 17115038 10.1038/nn1807 PMC1806686

[54] Larsen E, Liberman MC. Contralateral cochlear effects of ipsilateral damage: no evidence for interaural coupling. Hear Res. 2010;260: 70–80. 10.1016/j.heares.2009.11.011 19944141 PMC2815182

[55] Pennie RA. Prospective study of antibiotic prescribing for children. Can Fam Physician. 1998;44: 1850–56. 9789665 PMC2277846

[56] Deggouij N, Castelein S, Grégoire A, Laroche H, De Graeuwe C, de Toeuf C, et al Functional consequences of chronic ENT inflammation on the development of hearing and communicative abilities. B-ENT. 2012;19: 105–15. 23431614

[57] Joglekar S, Morita N, Cureoglu S, Schachern PA, Deroee AF, Tsuprun V, et al Cochlear pathology in human temporal bones with otitis media. Acta Otolaryngol. 2010;130: 472–6. 10.3109/00016480903311252 19895333 PMC2925651

[58] Jesic SD, Jotic AD, Babic BB. Predictors for sensorineural hearing loss in patients with tubotympanic otitis, cholesteatoma, and tympanic membrane retractions. Otol Neurotol. 2012;33: 934–40. 22722145 10.1097/MAO.0b013e318259b885

[59] Kolo ES, Salisu AD, Yaro AM, Nwaorgu OG. Sensorineural hearing loss in patients with chronic suppurative otitis media. Indian J Otolaryngol Head Neck Surg. 2012;64: 59–62. 10.1007/s12070-011-0251-5 23449378 PMC3244579

[60] Luntz M, Yehudai N, Haifler M, Sigal G, Most T. Risk factors for sensorineural hearing loss in chronic otitis media. Acta Otolaryngol. 2013;133: 1173–80. 10.3109/00016489.2013.814154 24125189

[61] Yehudai N, Most T, Luntz M. Risk factors for sensorineural hearing loss in pediatric chronic otitis media. Int J Pediatr Otorhinolaryngol. 2015;79: 26–30. 10.1016/j.ijporl.2014.10.025 25482507

[62] Katano H, Lino Y, Murakami Y, Kodera K. Temporal bone histopathology in a patient suspected of inner ear extension of otitis media. Nihon Jibiinkoka Gakkai Kaiho. 2005;108: 533–6. 15952341 10.3950/jibiinkoka.108.533

